# Topology Optimization Enables High-*Q* Metasurface for Color Selectivity

**DOI:** 10.1021/acs.nanolett.4c01858

**Published:** 2024-07-24

**Authors:** Huan-Teng Su, Lu-Yun Wang, Chih-Yao Hsu, Yun-Chien Wu, Chang-Yi Lin, Shu-Ming Chang, Yao-Wei Huang

**Affiliations:** Department of Photonics, College of Electrical and Computer Engineering, National Yang Ming Chiao Tung University, Hsinchu 300093, Taiwan

**Keywords:** metasurfaces, nonlocal, color selection, gratings, topology optimization

## Abstract

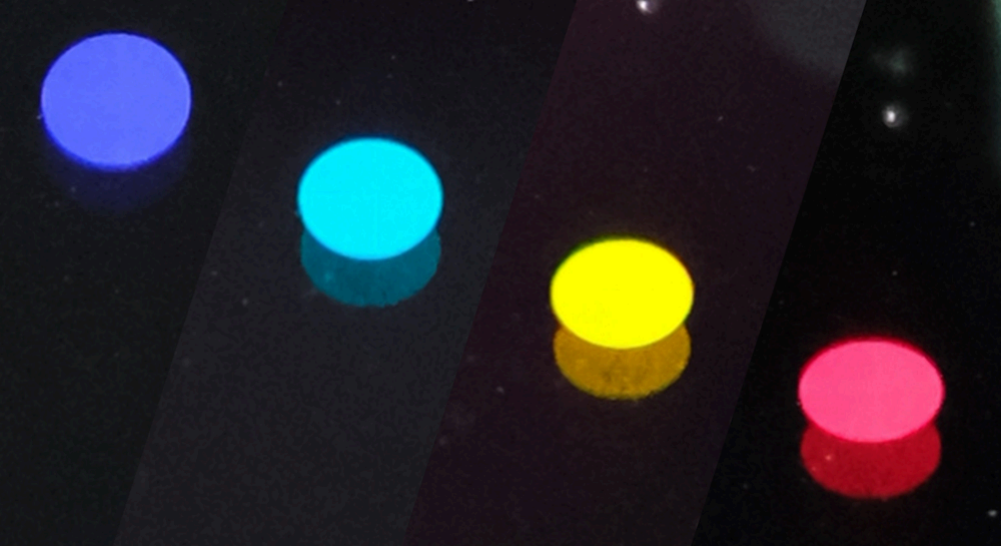

Nonlocal metasurfaces, exemplified by resonant waveguide gratings (RWGs), spatially and angularly configure optical wavefronts through narrow-band resonant modes, unlike the broad-band and broad-angle responses of local metasurfaces. However, forward design techniques for RWGs remain constrained at lower efficiency. Here, we present a topology-optimized metasurface resonant waveguide grating (MRWG) composed of titanium dioxide on a glass substrate capable of operating simultaneously at red, yellow, green, and blue wavelengths. Through adjoint-based topology optimization, while considering nonlocal effects, we significantly enhance its diffraction efficiency, achieving numerical efficiencies up to 78% and *Q*-factors as high as 1362. Experimentally, we demonstrated efficiencies of up to 59% with a *Q*-factor of 93. Additionally, we applied our topology-optimized metasurface to color selectivity, producing vivid colors at 4 narrow-band wavelengths. Our investigation represents a significant advancement in metasurface technology, with potential applications in see-through optical combiners and augmented reality platforms.

Metasurfaces are categorized into local and nonlocal based on their characteristics and behavior of building blocks.^1,2^ In the realm of metasurfaces, local and nonlocal variants exhibit distinct characteristics. Local metasurfaces shape the phase of incident light by referencing precomputed optical responses,^3−5^ which provides intuitive and convenient forward design of optics, such as metalenses,^6^ metasurface holograms,^7−9^ vortex lasers,^10^ etc. However, these devices often encounter limitations in spectral control. Due to the confinement of optical interactions within deeply subwavelength structures, they tend to exhibit broad-band characteristics, resulting in wavefront deformation across a wide frequency range. In contrast, nonlocal metasurfaces, such as guided-mode resonance gratings (RWGs),^11−13^ quasi-bound states in the continuum (q-BIC) structures,^14−16^ and perturbative structures,^17,18^ yield sharp spectral features. This is because guided-mode resonance gratings approximate the pure slab waveguide, enabling the excitation of multiple modes simultaneously at different wavelengths and resulting in higher quality factors (*Q*-factor). Additionally, q-BIC structures break the in-plane inversion symmetry through perturbations, leading to the creation of q-BIC with high yet finite *Q*-factors. However, traditional forward design approaches for RWGs and q-BIC structures often exhibit lower efficiency.^12,15^

RWGs defined based on their physical behavior rely on guided modes propagating over multiple gratings with geometrical continuity. RWGs consist of a waveguide and a dielectric grating. Due to the interaction between these two components, the exit spectrum exhibits many unique optical properties when light is incident on the device.^13^ The light, propagating in a single RWG, is diffracted out of the guide. Depending on the wavelengths, this leads to a very high reflection and a low transmission. Two RWGs consisting of two periods *U*_1_ and *U*_2_ have been demonstrated to achieve color-selective and versatile light steering.^12^ The schematic is shown in Figure 1c. Since there are two different periods, the outcoupled mode provides a different direction compared to that of incident light, while sharing the same guided modes inside the waveguide. By changing the period of the second RWG, the 2-RWG scheme filters portions of the spectrum ranges of the white light and redirects them in either the direct reflection or diffraction orders; however, they suffer in lower efficiency in the specific diffraction order. This challenge can potentially be addressed through inverse design, which starts with desired functionalities and optimizes geometries employing computational algorithms.^19,20^ Recent demonstrations of inverse design strategies of metasurface design include topology optimization,^21−24^ gradient based inverse design,^25^ genetic-type tree optimization,^26^ evolutionary optimization,^27^ deep learning,^28,29^ and hybrid combinations of these approaches. These techniques predominantly target enhancements in local metasurface characteristics. While deep learning can account for global features, it requires large data sets for construction. Although small-data learning^29^ methods operate with fewer data points, they still need highly characteristic data (nearly 20). Generating high-characteristic nanostructures corresponding to the spectral data is extremely challenging. In comparison, topology optimization does not require a pre-existing data set, as it optimizes based on gradient characteristics, making it more suitable. Such innovations underscore the transformative potential of inverse design in shaping high-*Q* and nonlocal metasurfaces, paving the way for diverse applications.

**Figure 1:**
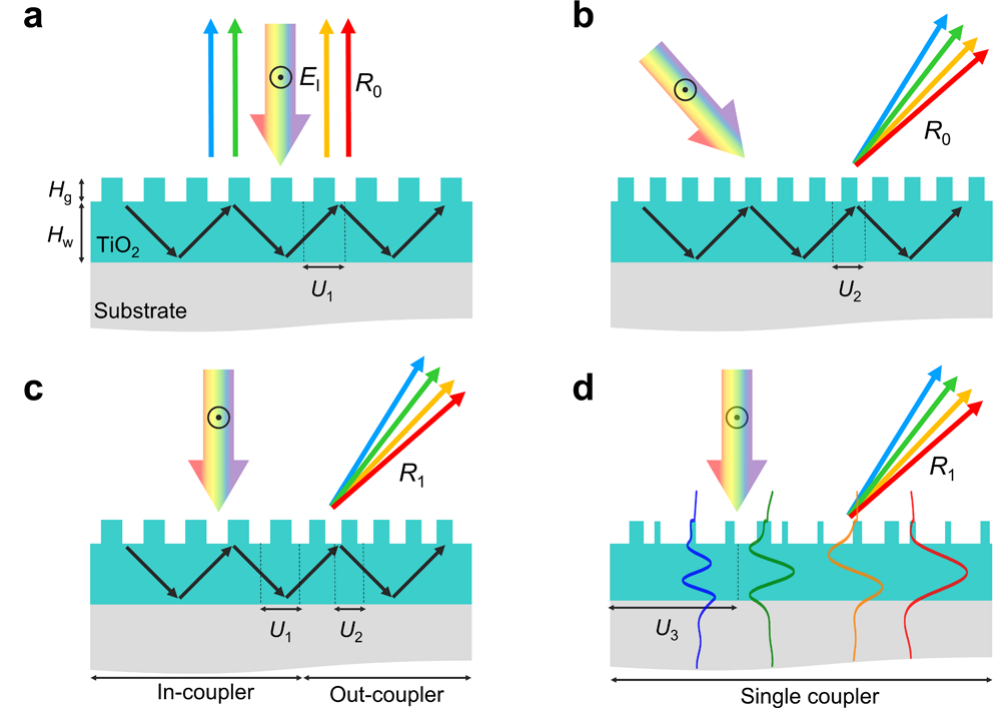
(a) Schematic of the normal diffracting RWG with a period of *U*_1_. (b) Schematic of the obliquely diffracting RWG with a period of *U*_2_. (c) Schematic of RWGs composed of in-coupler and out-coupler working in the visible spectrum. (d) Schematic of MRWG composed of single coupler working for both in- and out-coupling designed by using topology optimization.

In this study, we experimentally demonstrate a metasurface resonant waveguide grating (MRWG, as depicted in Figure 1d) characterized by outstanding color selectivity and redirection capabilities, along with high *Q*-factors that surpass traditional RWGs. Utilizing an adjoint-based topology optimization method, we incorporate various optimization strategies into the grating structure, resulting in superior performance. This method overcomes the limitations of conventional designs, eliminating the need for specific coupling regions and enabling full-area input and output coupling. This significantly enhances system flexibility and increases the cross-sectional area for more interactions, leading to higher diffraction efficiency and a superior *Q*-factor. Leveraging topological optimization allows us to optimize the grating structure while considering nonlocal effects, providing comprehensive control of light behavior and precise optical functionalities. With appropriate topological optimization strategies, our MRWG design demonstrates advantages in efficient color filtering and high *Q*-factors, making it suitable for applications such as biosensors, near-eye displays, and augmented reality, among others.

In the design process, the essential physics behind optimizations is necessary and helpful for progressing to the inverse design. First, we conducted simulations on two distinct types of RWGs: one with a period of *U*_1_ for normal incidence RWG (Figure 1a) and the other with a period of *U*_2_ for oblique incidence RWG (Figure 1b). During these simulations, we utilized variables such as *U*_1_*, U*_2_, waveguide height (*H*_w_ = 405 nm), and grating height (*H*_g_ = 160 nm) to ensure the overlap of their reflection spectra at resonance wavelengths (see Supporting Information S1). This strategy facilitates selective wavelength filtering, inducing narrow-band resonance effects. We chose to use the same *H*_w_ and *H*_g_ for both structures because it analogously aligns with the design approach in the literature (Figure 1c).^12^ These two structures can be fabricated together, and the two different gratings can share the same guided mode resonances (GMRs).

To enhance the diffraction efficiency, we follow the RWG structure illustrated in the forward method (Figure 1c), allocating half of the area to *U*_1_ and the remaining half to *U*_2_. However, the reported efficiency in the literature has remained below 10%.^12^ Therefore, to achieve higher efficiency, we utilize topology optimization (Figure 1d), allowing the entire area to contribute to both input and output coupling. In the meantime, the free-form structure is optimized to enhance diffraction efficiency at desired wavelengths.

The inverse design adopts the topology optimization method, where we initially calculated that the ideal grating period is designed as 870 nm (*U*_3_), which is an integer multiple of *U*_1_ and *U*_2_, generating only first-order diffraction in the visible spectrum. The physics behind this will be discussed in Figure 3b. Subsequently, we employed an adjoint-based topology optimization method for the grating, as illustrated in Figure 2a. We began with a random material distribution ρ(*x*) as pattern, which continuously varies from 0 to 1, linearly corresponding to relative permittivity values ranging from ε_Air_ to ε_TiO2_. Subsequently, we utilized the open source rigorous coupled-wave analysis (RCWA) package to perform the electromagnetic simulation of the grating,^30^ by setting parameters such as the angle of incidence, mode type, refractive index distribution of each layer, and geometric parameters of the structure. In a forward simulation, we calculated values including diffraction efficiency, the first-order complex amplitude, and forward electric field distribution at the grating. The first-order diffraction efficiency at a wavelength of 532 nm serves as the Figure of Merit (FoM) for this optimization. We then executed a backward simulation to reverse compute the adjoint electric field, using the phase of incidence determined by the first-order complex amplitude from the forward simulation. By utilizing both forward and adjoint electromagnetic simulations, we calculated the FoM gradient at each pixel of the MRWG: that is, ∇FoM. This gradient identifies perturbations in the pattern at pixel locations (ρ(*x*)) that enhance the overall FoM (see Supporting Information S2).

**Figure 2:**
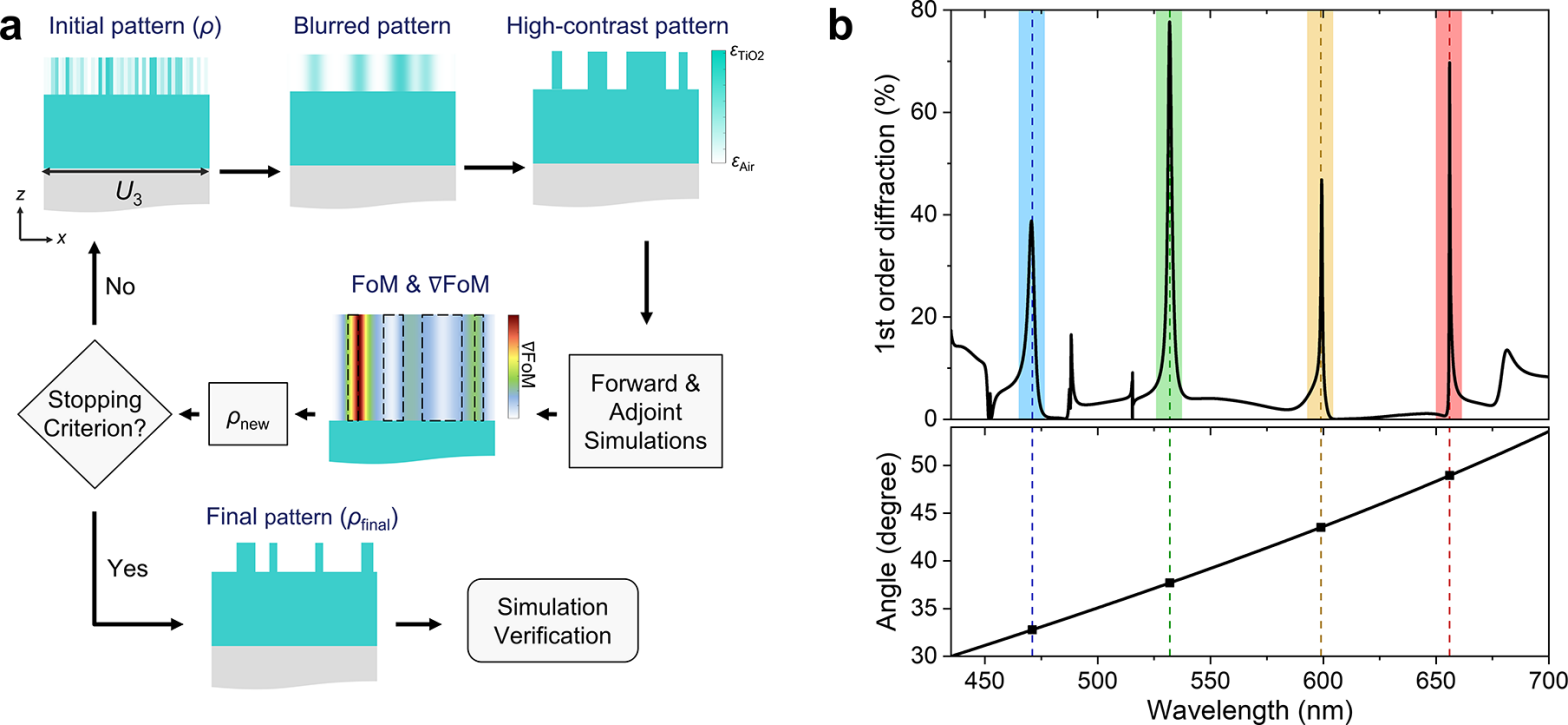
(a) Flowchart of the iterative optimization process. (b) Simulated first-order diffraction efficiency and angles of the MRWG. Colored regions represent the operational wavelengths for the four colors at λ_1_ = 471 nm, λ_2_ = 532 nm, λ_3_ = 599 nm, and λ_4_ = 656 nm.

The final step involves iteratively updating the pattern ρ^(*q*+1)^(*x*) with the FoM (ρ^(*q*)^) and its ∇FoM, where *q* indicates the *q*th iteration. Constraints are added to the optimization process to ensure that the final pattern converges to a binary design with only two relative permittivity values, ε_Air_ and ε_TiO2_.^21−23^ Initially, the values of pattern ρ range between 0 and 1. As the optimization iterations progress, these values tend to approach 0 or 1. This convergence can be achieved through a contrast function. Low contrast allows for greater freedom in the optimization process, but it may result in the final pattern not approaching values of 0 or 1, leading to mismatched binary patterns and low efficiency. Conversely, high contrast in the final pattern, with values approaching 0 or 1, can approximate a binary pattern, but it may also lead to a more difficult optimization process due to reduced freedom. Additionally, we consider the fabrication feasibility of the structure, which can be addressed by using a blur function with a specific radius. We determine a suitable blur radius to be 28 nm, corresponding to a blur dimension or line width of approximately 54 nm, matching our fabrication limitations. The aspect ratio of the meta-atoms reaches about 3, which is smaller than the typical aspect ratio of nanopillars, ranging from 5 to 8. A larger blur radius reduces the geometric freedom and the potential for higher efficiency, whereas a smaller radius results in more complex fabrication processes.

We validated the optimized MRWG by examining the simulation results. The diffraction efficiency is depicted in Figure 2b. The incident light is illuminated normally, covering a range of visible wavelengths. It is observed that peak values occur at wavelengths of 471, 532, 599, and 656 nm, respectively. This indicates that the MRWG successfully reflects blue, green, yellow, and red light within the visible spectrum. The full width at half-maximum (fwhm) of the four peaks ranges from 0.48 to 3.59 nm. Our MRWG, designed with topology optimization, significantly increases efficiency, reaching up to 77.7% (for additional analyses, refer to Supporting Information S3–S6). Without using topology optimization, the efficiency reported in the literature is less than 10%.^12^ Furthermore, the *Q*-factor is determined by the guided modes, and our results show very high *Q*-factors ranging from 131 to 1367; detailed values are presented in Table 1.

**Table 1:** Simulation Numerical Statistics

	wavelength (nm)	efficiency (%)	fwhm (nm)	*Q*-factor	diffraction angle (deg)
λ_1_	471	38.8	3.59	131	32.8
λ_2_	532	77.7	2.25	236	37.7
λ_3_	599	46.8	0.952	630	43.6
λ_4_	656	69.7	0.482	1362	49.0

We employed a momentum analysis approach to investigate the resonance wavelength phenomenon in the MRWG structure. To begin, we take the resonance at red light (λ_4_) for example, with the physical phenomena extendable to other wavelengths. The transverse momentum of light coupling into the waveguide can be obtained by the momentum conservation equation for in-coupling (eq 1), which is
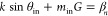
1

Here, *k* represents the wavenumber, β_*n*_ is the transverse momentum, θ_in_ is the angle of incidence, and *G* denotes the grating momentum, which is 2π/*U*_3_. When light is normal incident to the MRWG, the transverse momentum from the light is zero. The momentum coupled inside the waveguide is then determined by *G* with *m*_in_ times.

Additionally, the β_*n*_ of light coupling out from the waveguide can be obtained through the momentum conservation equation for out-coupling (eq 2), which is
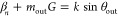
2

The diffraction angle θ_out_ is then estimated, where the momentum *k* sin θ_out_ is influenced by adding β_*n*_ with *m*_out_ times *G*. Figure 3a,b visualizes the momentum conservation process described above. The values of (*m*_in_, *m*_out_) are (−3, 4), where a negative value indicates a negative direction or momentum as the guided mode propagates inside the waveguide (β_*n*_ = −3*G*). In Figure 3b, it can be observed that the −3*G* momentum corresponds to a specific mode number (*n*th) at wavelengths ranging from λ_4_ to λ_1_. Similarly, when coupling out of the waveguide, the light experiences a 4*G* momentum into the air layer, leading to a positive diffraction angle (θ_out_) as determined by eq 2. This structure also provides the possibility values of (*m*_in_, *m*_out_) as (4, −3), but with much lower efficiency, e.g., resonances at 452, 488, and 515 nm as shown in Figure 2b. This case has 4*G* momentum propagating inside the waveguide (details in Supporting Information S3).

**Figure 3:**
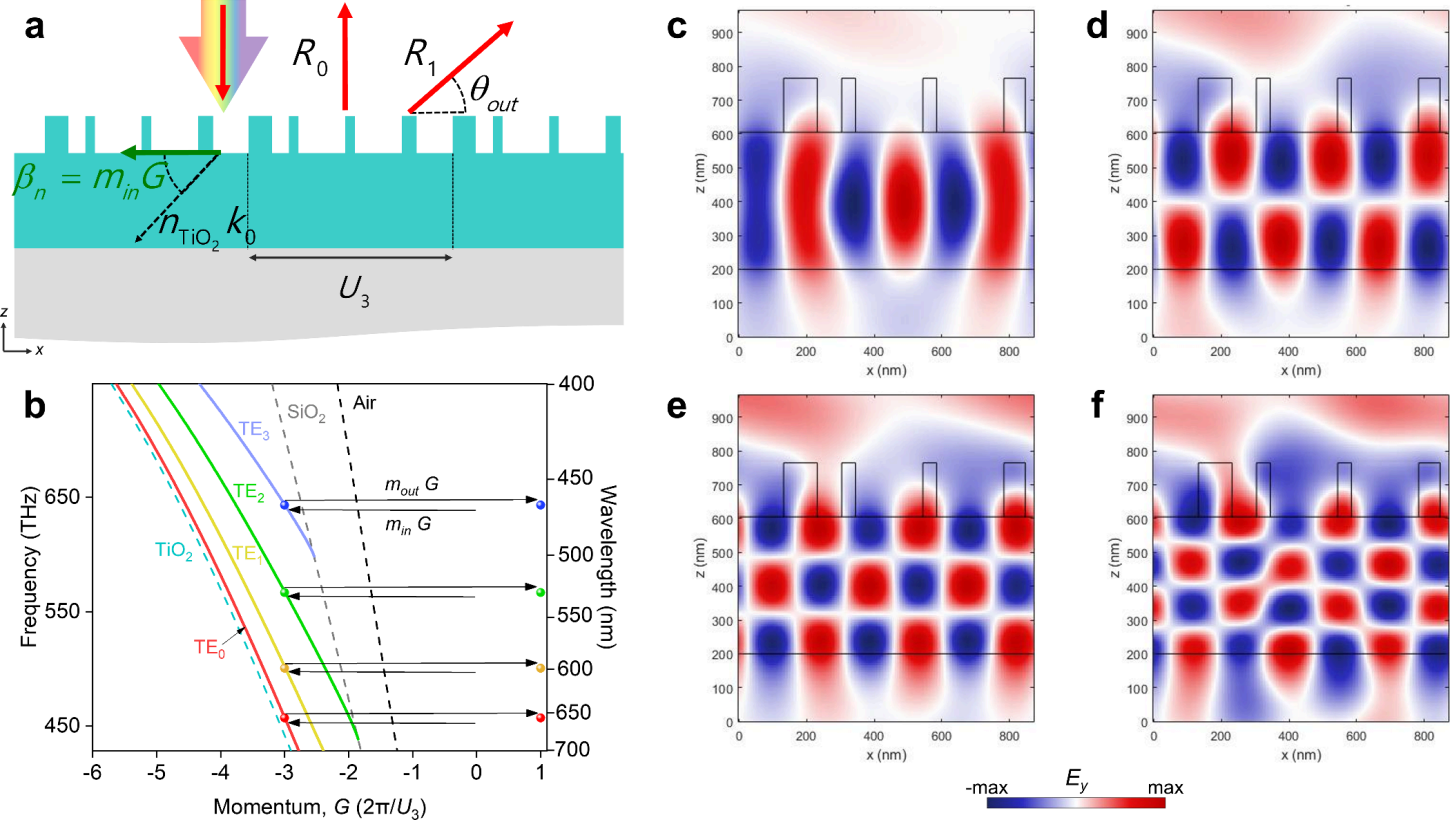
(a) Schematic representation of the momentum relationship when light is incident on the MRWG with a *U*_3_ period. (b) Modal relation for the waveguide modes supported by the TiO_2_ waveguide. The black, gray, and cyan dashed curves are the light lines for Air, SiO_2_ and TiO_2_, respectively. The horizontal arrow to the left indicates the excitation of the grating by a normally incident planewave. The arrow to the right designates the decoupling from the TE_*n*_ waveguide mode into the first diffracted order. *U*_3_ is the grating period. (c–f) Electric field (*E*_y_) of waveguide resonance effects: (c) fundamental mode (TE_0_) at a wavelength of 656 nm; (d) second mode (TE_1_) at a wavelength of 599 nm; (e) third (TE_2_) mode at a wavelength of 532 nm; (f) fourth mode (TE_3_) at a wavelength of 470 nm.

The significance of values 3 and 4 is linked to the relationship of 3*U*_1_ = 4*U*_2_. In other words, initially, *U*_3_ = |*m*_in_|*U*_1_ = |*m*_out_|*U*_2_. Therefore, the design principle of the MRWG, such as momentum conservation while coupling in and out of the structure, is determined by the coupling momentum coefficients (*m*_in_, *m*_out_).

Ultimately, we utilized Figure 3b to identify the resonance wavelengths corresponding to the mode number (*n*th) when β_*n*_ = −3*G*. This process resulted in obtaining cross-sectional images of four resonance mode numbers from Figure 3c–f. The electric field exhibits 0 (1, 2, 3) node(s) along the *z*-direction, corresponding to the TE_0_ (TE_1_, TE_2_, TE_3_) waveguide modes. In other words, β_1_–β_4_ at λ_4_–λ_1_ correspond to quantized waveguide modes.

We fabricated our MRWG sample using electron-beam lithography, atomic layer deposition, and the high-density plasma reactive ion etching (HDP-RIE) method (see Supporting Information S7 for detailed manufacturing procedures). Figure 4a shows the scanning electron microscope (SEM) image of our fabricated sample, which includes ∼3 periods with a structure distribution consistent with the MRWG. The tiled SEM image shown in the inset of Figure 4a indicates a grating height of about 160 nm. Subsequently, we conducted spectral measurements of the first-order reflective diffraction efficiency as a function of wavelength using our experimental setup (see Supporting Information S8 for details), as depicted in Figure 4b. In this spectrum, red lines represent the experimental results. Four distinct peaks can be observed. It is worth noting that the efficiency is particularly high (59.1%) in the blue spectral region, with a relatively high *Q*-factor as high as 93.2. Detailed experimental values of the MRWG are presented in Supporting Information Table S1.

**Figure 4:**
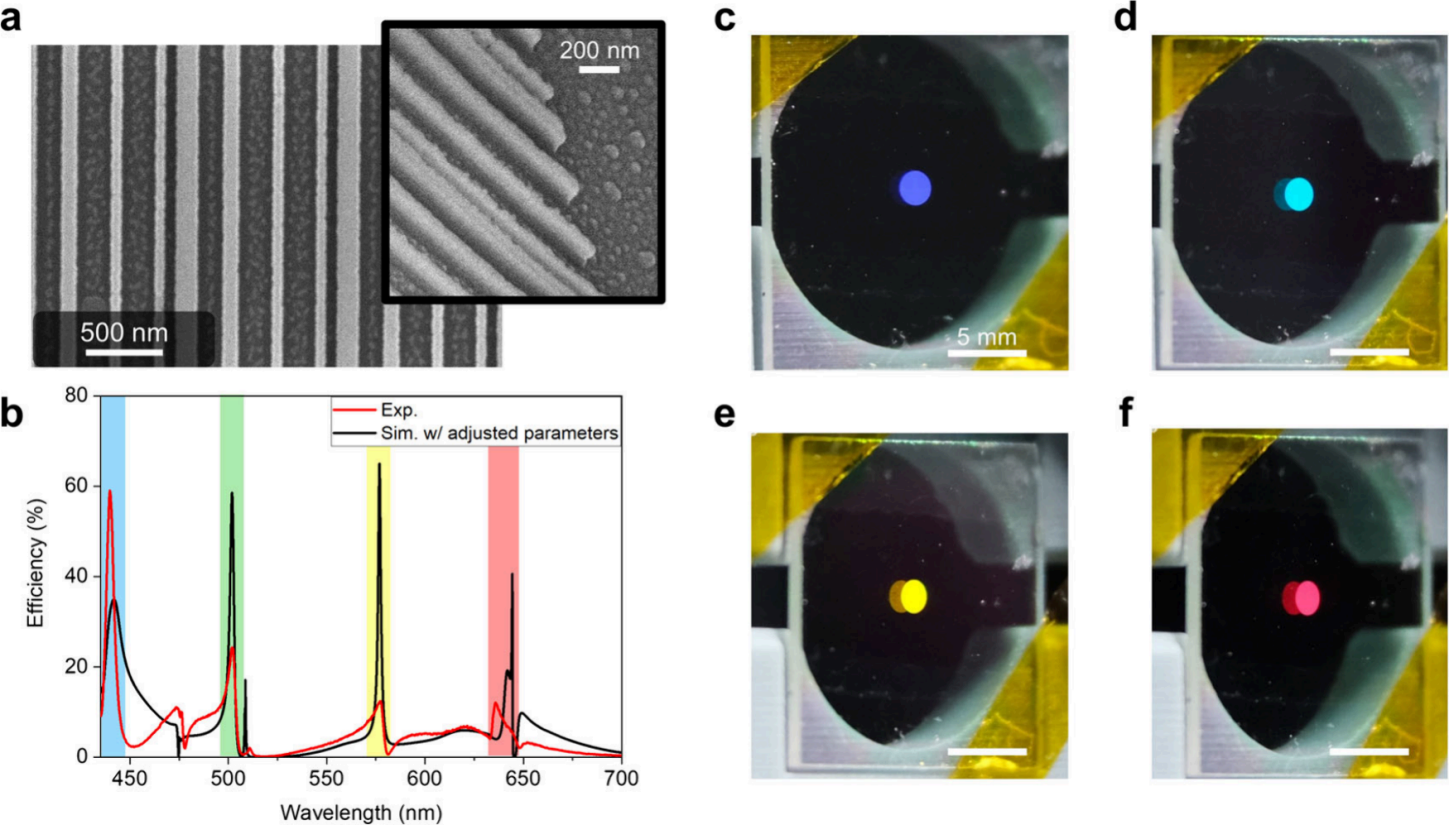
(a) SEM image displaying the MRWG sample, with an inset showing the 45° tiled view of a portion of the sample. (b) Comparison of measured spectrum of MRWG (red line) and simulated spectrum with adjusted parameters (black line). Colored regions highlight the good alignment of peak wavelengths. (c–f) MRWG reflection images captured using a microfocus camera.

However, we observe a blue shift in the experimentally measured peaks compared to the optimization results along with relatively lower efficiency. After investigating structural and refractive index inaccuracies, we found that this result could be attributed to process inaccuracies and TiO_2_ refractive index discrepancies (see Supporting Information S8). These factors led to a lower efficiency and a blue shifting of the peaks. Figure 4b shows a good alignment of peak wavelengths in the simulated spectrum with adjusted parameters (black line) and in the experimental spectrum (red line). The simulated spectrum indicates a lower refractive index for TiO_2_ and overetched waveguides in the experiments.

Figure 4c–f displays images of the MRWG captured using a microfocus camera. We positioned the camera at specific angles corresponding to the 4 diffraction peaks and captured images accordingly. This process required precise alignment of the camera at specific angles to observe and capture optical effects. In these captured images, from left to right, we have the reflection effects in different colors for blue, cyan (blue shift of green), yellow, and red light. Different colors represent the diffraction peak of MRWG at 4 designed wavelengths, and these effects are only visible at specific angles, further illustrating the color-selective properties of the MRWG.

In this study, we experimentally demonstrate a novel MRWG that utilizes adjoint-based topology optimization to achieve remarkable color selectivity, redirection capabilities, and high *Q*-factors. Effectively overcoming the limitations of traditional RWGs, such as specific coupling regions, our MRWG design provides a full area for both input and output coupling, increasing flexibility and interaction area for enhanced overall efficiency and *Q*-factors. Incorporating nonlocal effects into the topology optimization process enables precise control of the light behavior, allowing efficient color filtering and high *Q*-factors. The MRWG design successfully diffracts blue, green, yellow, and red light beams with peak efficiency, as observed in experimental results. Despite minor discrepancies between experimental and simulation results due to process inaccuracies and unsatisfactory refractive index, the MRWG’s color-selective properties are clearly demonstrated, offering promising opportunities for various optical applications. In summary, the MRWG design presented in this study represents a significant advancement in metasurface technology, showcasing its potential for practical and efficient color selectivity and redirection. Additionally, for one-dimensional optimization, the FoM was set for a single wavelength, resulting in 4 high-*Q* wavelengths being optimized simultaneously. This occurs because the low degrees of freedom in 1D optimization tend to couple out resonance wavelength. In the future, if there is a need to depress a specific wavelength or select only one wavelength, more structural freedom, such as 2D optimization, will be necessary. Our MRWG is suitable for a wide range of applications, including biosensors,^31^ genetic screening,^32^ near-eye displays,^33^ and augmented reality.^34,35^ This research contributes to the ongoing development of metasurfaces with improved performance in spectrum engineering and diverse applications in narrow-band nanophotonics.

## Supporting Information Available

Additional details of initial forward design, iterative optimization process, simulations of different incidence in TE and TM modes, fabrication process, optical setup, and experimental results (PDF)
