# Supplementary material for: Topology Optimization Enables High-Q Metasurface for Color Selectivity
Source: Nano Lett. 2024 Jul 24;24(33):10055–61. doi: 10.1021/acs.nanolett.4c01858 (PMC11342354; doi:10.1021/acs.nanolett.4c01858)
Supplement: Supplementary file 1 — nl4c01858_si_001.pdf [file nl4c01858_si_001.pdf]

Supporting Information for

Topology Optimization Enables high-*Q* Metasurface for Color Selectivity

Huan-Teng Su,[†] Lu-Yun Wang,[†] Chih-Yao Hsu,[†] Yun-Chien Wu,[†] Chang-Yi Lin,[†] Shu-Ming Chang,[†] and Yao-Wei Huang[†,*]

[†] Department of Photonics, College of Electrical and Computer Engineering, National Yang Ming Chiao Tung University, Hsinchu 300093, Taiwan

*Address correspondence to: ywh@nycu.edu.tw

Contents

S1. Initial forward design

S2. Iterative optimization process

S3. Simulations of top incidence (TE mode)

S4. Simulations of bottom incidence (TE mode)

S5. Simulations of oblique incidence (TE mode)

S6. Simulations of top incidence (TM mode)

S7. Fabrication process

S8. Optical setup and experimental results

S1. Initial forward design

In the forward design, we conducted simulations of reflection spectra on two distinct types of RWGs. The first type had a period of U_1, tailored for normal incidence RWG (Figure S1a), while the second type featured a period of U_2, specifically designed for oblique incidence RWG (Figure S1b). Throughout these simulations, we explored various numerical values for H_w, H_g, U_1, and U_2 with the objective of achieving selective wavelength filtering. Ensuring the overlap of their reflection spectra at resonance wavelengths was crucial, as illustrated in Figure S1c. This meticulous design approach allowed us to implement wavelength filtering ($3U_1 = 4U_2 = U_3$).

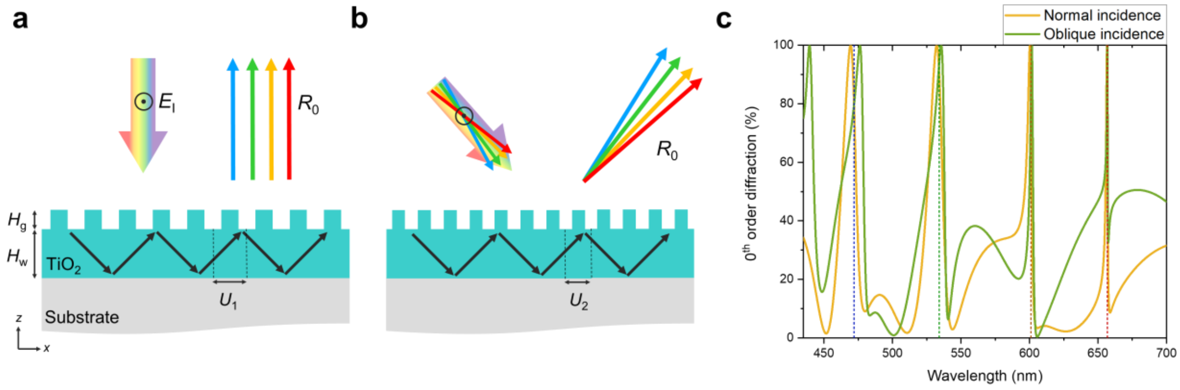

Figure S1. (a) Schematic of the normal diffracting RWG with a period U_1. (b) Schematic of the obliquely diffracting RWG with a period U_2. (c) Schematic illustrating the reflection spectra of RWGs with periods U_1 (yellow line) and U_2 (green line) is shown.

S2. Iterative optimization process

Initially, we computed the optimal grating period as 870 nm (U_3), an integer multiple of U_1 and U_2, resulting in the 1st diffraction within the visible spectrum. Subsequently, we employed an adjoint-based topology optimization method for grating design, where the flowchart is shown in Figure S2. We generated a random structure distribution ranging from 0 to 1, corresponding to the range from $\varepsilon_{\mathrm{Air}}$ to $\varepsilon_{\mathrm{TiO2}}$, which we used to form the grating (denoted as ρ_1). We then applied blur function to ρ_1 to create a continuous distribution between 0 and 1 (ρ_2), as shown in Eq. S1a:

$$\rho_2 = \mathbf{M}\rho_1, \tag{S1a}$$

$$\mathbf{M} = \begin{pmatrix} M_{11} & M_{12} & \cdots & M_{1N} \\ M_{21} & M_{22} & \cdots & M_{2N} \\ \vdots & \vdots & \ddots & \vdots \\ M_{N1} & M_{N2} & \cdots & M_{NN} \end{pmatrix} = \begin{pmatrix} w_0 & w_1 & \cdots & w_{N/2-1} & w_{-N/2} & \cdots & w_{-1} \\ w_{-1} & w_0 & \cdots & w_{N/2-2} & w_{N/2-1} & \cdots & w_{-2} \\ \vdots & \vdots & \ddots & \vdots & \vdots & \ddots & \vdots \\ w_1 & w_2 & \cdots & w_{-N/2} & w_{-N/2+1} & \cdots & w_0 \end{pmatrix}, \tag{S1b}$$

$$w_j = \frac{\exp\left[-\frac{\left(x_j - x_0\right)^2}{b^2}\right]}{\sum_{j=-N/2}^{N/2-1} \exp\left[-\frac{\left(x_j - x_0\right)^2}{b^2}\right]}. \tag{S1c}$$

Here, matrix $\mathbf{M}$ is an $N \times N$ matrix where N is 192 in our case representing the grating period U_3. Each row of $\mathbf{M}$ is a cyclic shift of the normalized Gaussian weight vector $\mathbf{w}$ (Eq. S1b). To calculate each element of $\mathbf{w}$ vector (shown in Eq. S1c), x_j is the position of the pixel, x_0 is the mean of the Gaussian distribution, j is an even integer from -N/2 to N/2-1, and b is the standard deviation parameter of the Gaussian distribution representing the blur radius.

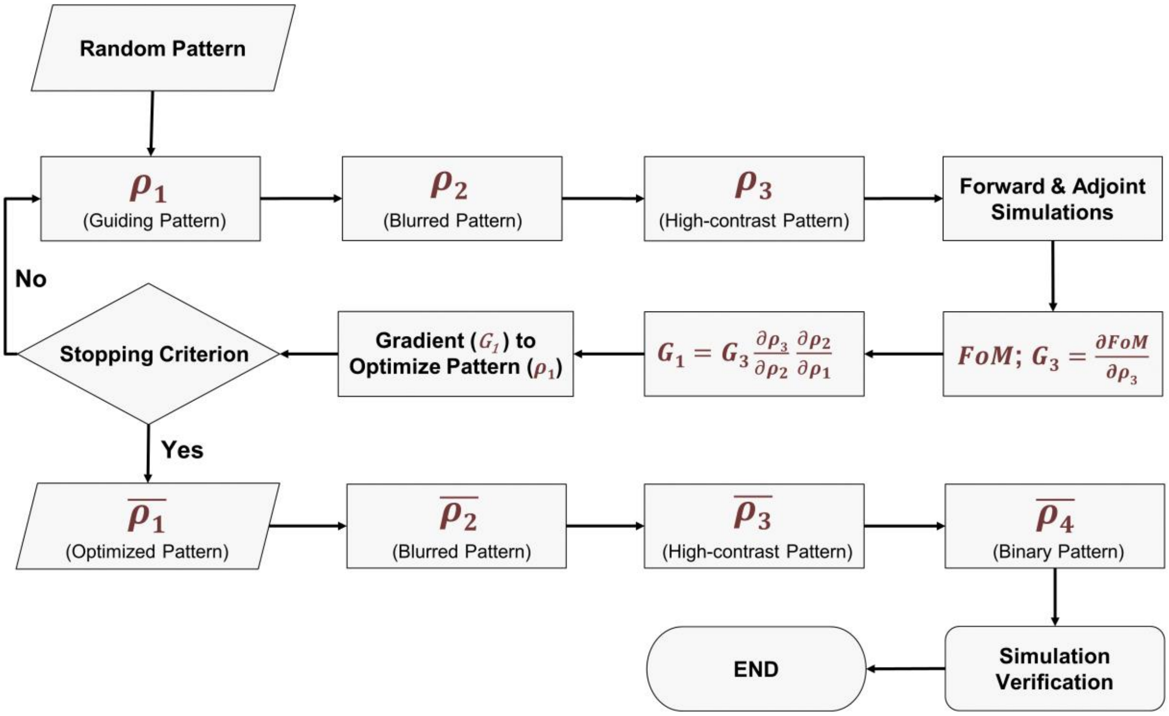

Figure S2. Flowchart of the iterative optimization process.

We then applied a contrast function to ρ_2 to enhance values closer to 0 or 1 (ρ_3). The function is shown in Eq. S2:

$$\rho_3 = \frac{\tanh(\beta \times \gamma) + \tanh\left[(\rho_2 - \gamma)\beta\right]}{\tanh(\beta \times \gamma) + \tanh\left[(1-\gamma)\beta\right]}. \tag{S2}$$

Here, the γ represents the midpoint of the binary distribution, usually equal to 0.5, the β denotes the magnitude of the contrast, which is set as 50 in our optimization.

Next, we conducted forward simulations by using ρ_3 to calculate various physical parameters such as forward electric field ($\mathbf{E}(x)$), the 1st diffraction coefficient (r_{1st}), and the 1st diffraction efficiency (serving as Figure of Merit (FoM) for optimization). We utilized the open source rigorous coupled-wave analysis (RCWA) package to perform the 1st diffraction efficiency of the grating [30].

Subsequently, we performed reverse simulations to compute the adjoint electric field ($\mathbf{E}_{\text{adjoint}}(x)$), utilizing the phase information obtained from the forward simulations. Combining forward and adjoint electromagnetic field simulations, we calculated the $\partial \text{FoM}/\partial \rho_3$ denoted as G_3 as: [21]

$$G_3 = \left.\frac{\partial \text{FoM}}{\partial \rho_3}\right|_x \propto \text{Re}\left[conj\left(r_{1st}\right)\mathbf{E}\left(x\right) \bullet \mathbf{E}_{\text{adjoint}}\left(x\right)\right] \tag{S3}$$

The chain rule was then applied to derive the $\partial \text{FoM}/\partial \rho_1$ (denoted as G_1) relative to the initial dielectric constant ρ_1.

$$G_1 = \frac{\partial \text{FoM}}{\partial \rho_1} = G_3 \frac{\partial \rho_3}{\partial \rho_2}\frac{\partial \rho_2}{\partial \rho_1} \tag{S4}$$

We iteratively updated $\rho_1^{(q+1)}(x)$ by utilizing G_1, where q represents the qth iteration. As optimization progressed, convergence towards 0 or 1 was facilitated by the contrast function. Additionally, we considered the feasibility of structure fabrication, addressing it with a blur function of specific radius.

Ultimately, upon finding the optimal ρ_1 solution (denoted as $\overline{\rho_1}$), we applied blur function and contrast function to find $\overline{\rho_2}$ and $\overline{\rho_3}$. Additional binarization was applied to create a structure containing only 0 and 1 as $\overline{\rho_4}$. Figure S3a shows the optimal solution of one optimization. And Figure S3b shows the increase of the FoM (the 1st order diffraction efficiency) with the iterations. One typical optimization run takes approximately 5 to 10 minutes, involving about 100 to 150 iterations. Finally, we conducted simulation analysis to validate this structure.

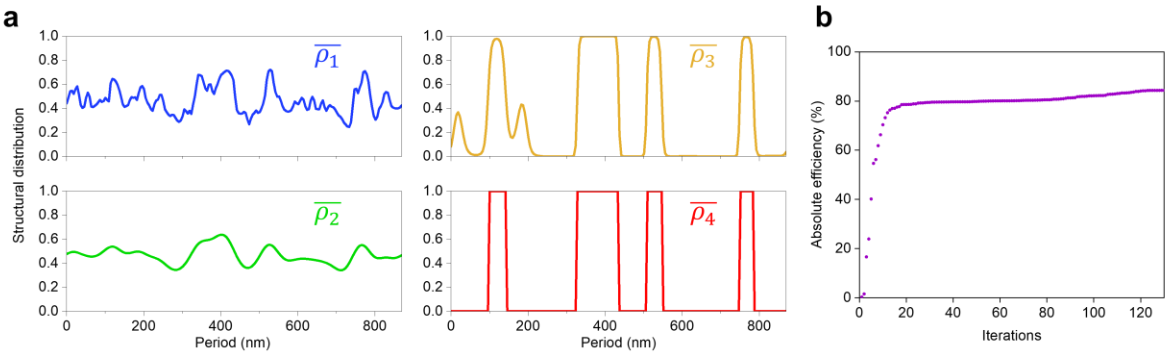

Figure S3. (a) Optimal structure distribution. (b) Diffraction efficiency (FoM) over the optimization iteration.

S3. Simulations of top incidence (TE mode)

Simulations were conducted to analyze top incidence (TE mode) regarding guide mode resonance in the MRWG structure (Figure S4a). The results revealed prominent 1st order reflective diffraction at four specific wavelengths, accompanied by low 0th order transmission. Following this observation, optimization focused on enhancing the 1st order reflection while suppressing the 0th and negative 1st order reflections to enhance efficiency and prevent interference with other optical functionalities (Figure S4b). Notably, the transmission efficiency diagram for the 0th order shows lower values within a specific wavelength range, attributed to waveguide mode resonance effects at that wavelength. The wavelength range with high reflection efficiency corresponds to positions where 0th order transmission efficiency is low (Figure S4c). Conversely, the wavelength range with low reflection efficiency aligns with positions where 0th order transmission efficiency is high.

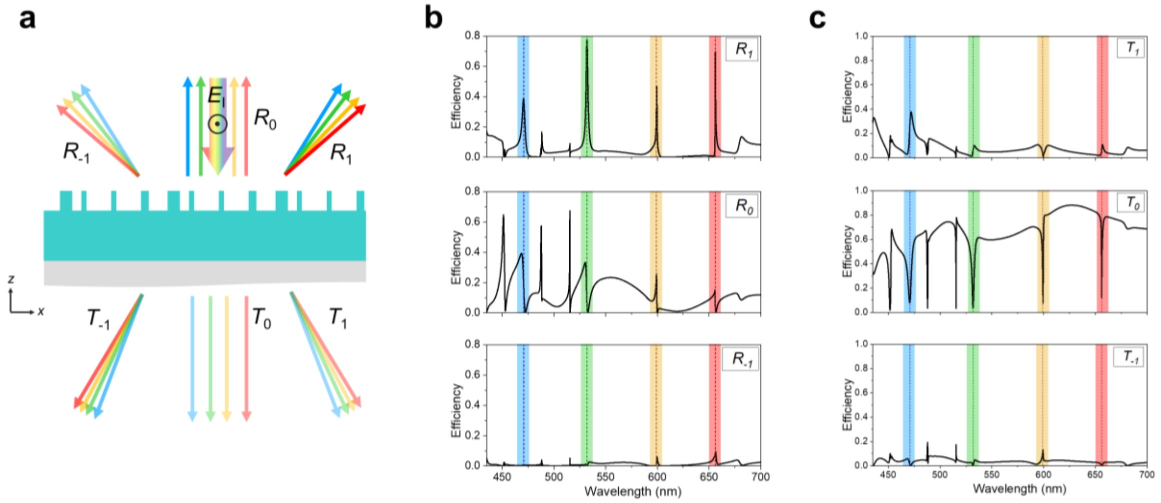

Figure S4. (a) Schematic of the normal incident TE mode MRWG. (b) Schematic of the reflective diffraction spectra of MRWG. (c) Schematic of the transmissive diffraction spectra of MRWG.

Figure S5a shows the R_1 diffraction spectrum with different momenta of incidence ($k_{in} = k \sin\theta_{in}$) corresponding to various angles of incidence. The peak frequencies of diffraction efficiency

change (increase or decrease) with the angle of incidence, indicating the band structure of the MRWG. These frequency shifts represent different coupling schemes with the waveguide. By comparing this with the dispersion of the TiO_2 waveguide mode (Figure S5b), we find that these peak values of diffraction efficiency align well with the waveguide's dispersion. For instance, when (m_{in}, m_{out}) = (-3, 4), a negative value of m_{in} indicates a negative direction or momentum as the guided mode propagates inside the waveguide ($\beta_n = k_{in} - 3G$). In another scenario, (m_{in}, m_{out}) = (4, -3), with $k_{in} + 4G$ momentum propagating inside the waveguide, indicates a positive direction or momentum. These two coupling schemes match well with the frequencies of the peak values of diffraction efficiency. Figure S5c is the R_1 diffraction spectrum, where the peak frequencies match well with the frequencies of the TE_n momenta at $k_{out} = 1G = k \sin\theta_{out}$ shown in Figure S5b.

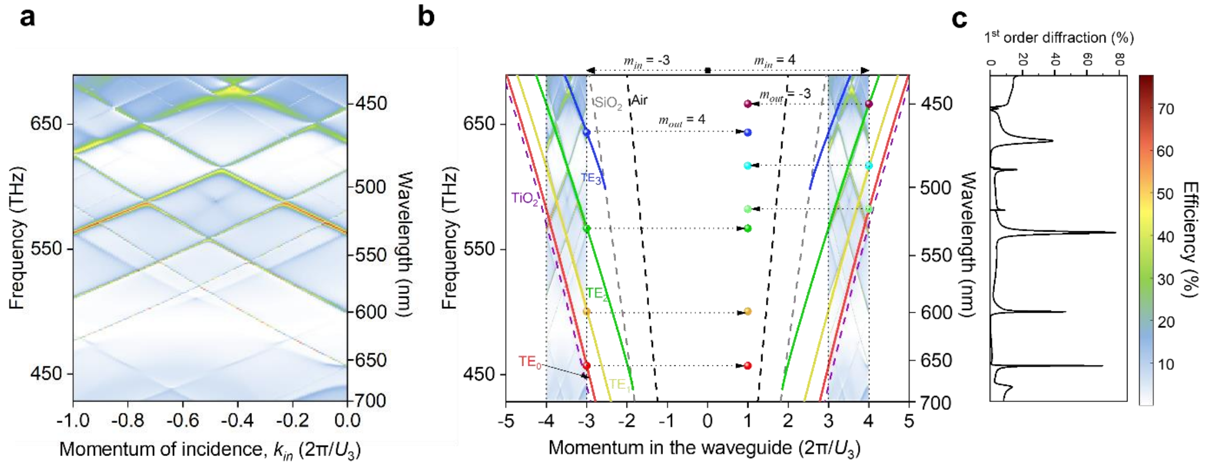

Figure S5. (a) Schematic R_1 diffraction spectrum versus momentum of incidence $k \sin\theta_{in}$. (b) Comparison of (a) with the modal relation for the waveguide modes supported by the TiO_2 waveguide. The black, grey, and purple dashed curves represent the light lines for air, SiO_2, and TiO_2, respectively.

S4. Simulations of bottom incidence (TE mode)

Simulations were conducted to analyze bottom incidence (TE mode) regarding guide mode resonance in the MRWG structure (Figure S6a). The results showed significant 0th order reflective diffraction (Figure S6b) at 4 specific wavelengths, coupled with low 0th order transmission (Figure S6c) at these wavelengths. Notably, the reflection diagram exhibited high efficiency at particular wavelengths, contrasting with the transmission diagram where low efficiency was observed for the same wavelengths. This suggests that, in a simulated scenario simulating post-application to augmented reality (AR) glasses, most light would transmit through the glasses, allowing wearers to perceive the external environment with minimal disruption. However, wavelengths demonstrating strong reflection could be selectively targeted and supplemented with narrow-band laser light sources to further enhance visual experiences.

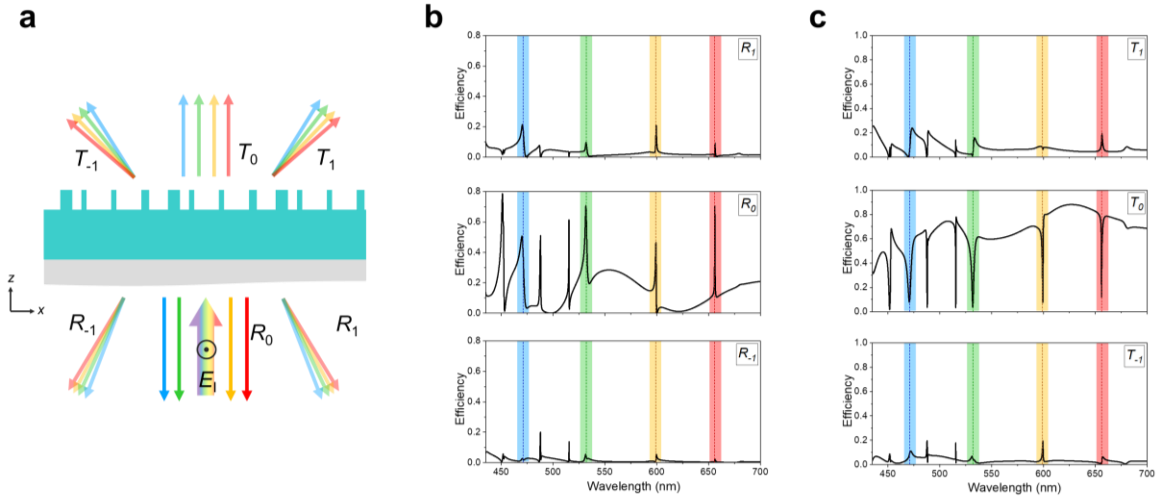

Figure S6. (a) Schematic of the normal bottom incidence TE mode MRWG. (b) Schematic of the reflective diffraction spectra of MRWG. (c) Schematic of the transmissive diffraction spectra of MRWG.

S5. Simulations of oblique incidence (TE mode)

Simulations were conducted to analyze oblique incidence (TE mode) regarding guide mode resonance in the MRWG structure (Figure S7a). Revealing high 1st order reflective diffraction (Figure S7b) at 4 specific wavelengths and low 0th order transmission (Figure S5c) at these wavelengths.

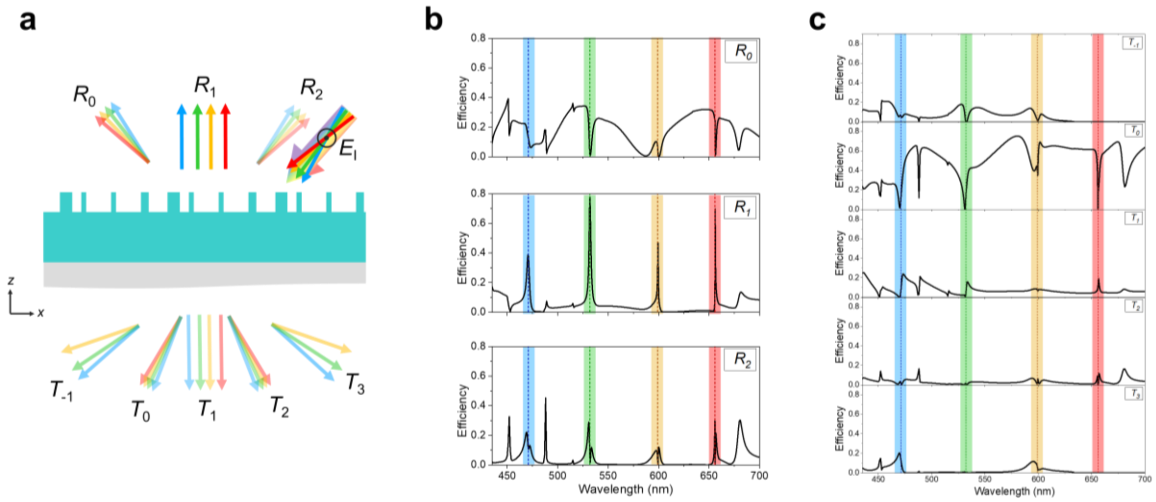

Figure S7. (a) Schematic of the oblique incidence TE mode MRWG. (b) Schematic of the reflective diffraction spectra of MRWG. (c) Schematic of the transmissive diffraction spectra of MRWG.

We plan to further explore the potential application of a laser light source incident on AR glasses at specific angles to interact with the MRWG. When a laser light source, containing primary colors, is directed at the RWMG at an angle, the 1st order reflection returns to the observer's eyes, creating a virtual image. This method allows for the manipulation of color and imaging, enhancing the virtual experience. The 0th order direct transmission efficiency is significantly low after the laser light source is incident. The majority of light passes through the glasses, creating an internal image and minimizing the external intensity. This design ensures that

individuals other than the wearer find it challenging to observe the internal image generated by the light source, thereby safeguarding user privacy and information security.

S6. Simulations of top incidence (TM mode)

In simulations focusing on the TM mode (Figure S8a), it is evident that the reflective diffraction efficiency is low (Figure S8b). It is essential to note that the optimization primarily targets the TE mode. Therefore, the observed low efficiency in the TM mode is a direct consequence of the optimization being tailored for the TE mode. To enhance the effectiveness of the TM mode, there is a need to increase the thickness of the waveguide layer.

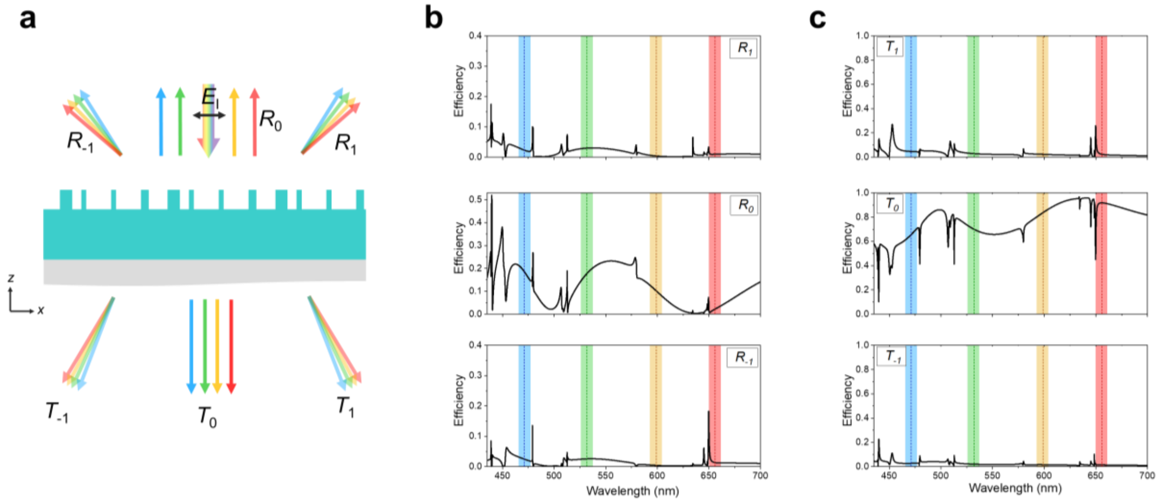

Figure S8. (a) Schematic of the normal incident TM mode MRWG. (b) Schematic of the reflective diffraction spectra of MRWG. (c) Schematic of the transmissive diffraction spectra of MRWG.

S7. Fabrication process

To achieve high aspect-ratio TiO_2 meta-atoms with minimal surface roughness for optimal optical properties (low scattering), we employ a bottom-up fabrication process based on atomic layer deposition (ALD), as illustrated in Figure S9. Our design involves deposition of a 405-nm-thick layer of TiO_2 film on a fused silica substrate, covering an area of 1 × 1 cm^2. The fabrication process begins by spin-coating electron beam resist (EBR) onto the substrate, with a thickness (t_{resist}) of 160 nm, as shown in Figure S9a. To enhance the uniformity of electron beam lithography (EBL) exposure, a thin layer of conductive resin (Espacer Z300) is spin-coated on top of the EBR before exposure. Next, the EBR layer is patterned using an EBL system (VOYAGER, Raith) and subsequently developed in a solvent to dissolve the EBR in the exposed region. The remaining EBR forms the inverse of our designed structure (Figure S9b). The developed sample is then moved into the ALD chamber, where the deposition is carried out at a temperature of 90°C. This temperature is chosen to maintain the amorphous phase of TiO_2, as crystalline TiO_2 tends to have more pronounced grain boundaries, leading to greater power loss due to increased surface scattering. The low temperature also prevents the EBR from cracking or collapsing during ALD deposition. During ALD operation, gaseous TiO_2 precursors, TDMAT, and H_2O are repeatedly inserted into the ALD chamber. The deposition continues until TiO_2 completely fills up the EBR holes. Due to the isotropic deposition mechanism of ALD, the total coating thickness requires at least $w_{max}/2$ (Figure S9c), where w_{max} is the maximum width of all EBR gaps and the largest width of designed meta-atoms. After deposition, the excess TiO_2 film overlaying on the top of the EBR and TiO_2 patern is removed by high-density plasma reactive ion etching (HDP-RIE) (Figure S9d). Since the surface is nearly planar after ALD, considerations such as etching selectivity between the etching mask and TiO_2 or the etching verticality of meta-atoms are minimized. The etching

process is simplified, focusing on determining the etching depth of the extra TiO_2 film, which is approximately equal to the measured thickness of the deposition using an ellipsometer. Finally, the remaining EBR and other residual nanostructures are removed, resulting in the completion of our MRWG (Figure S9e).

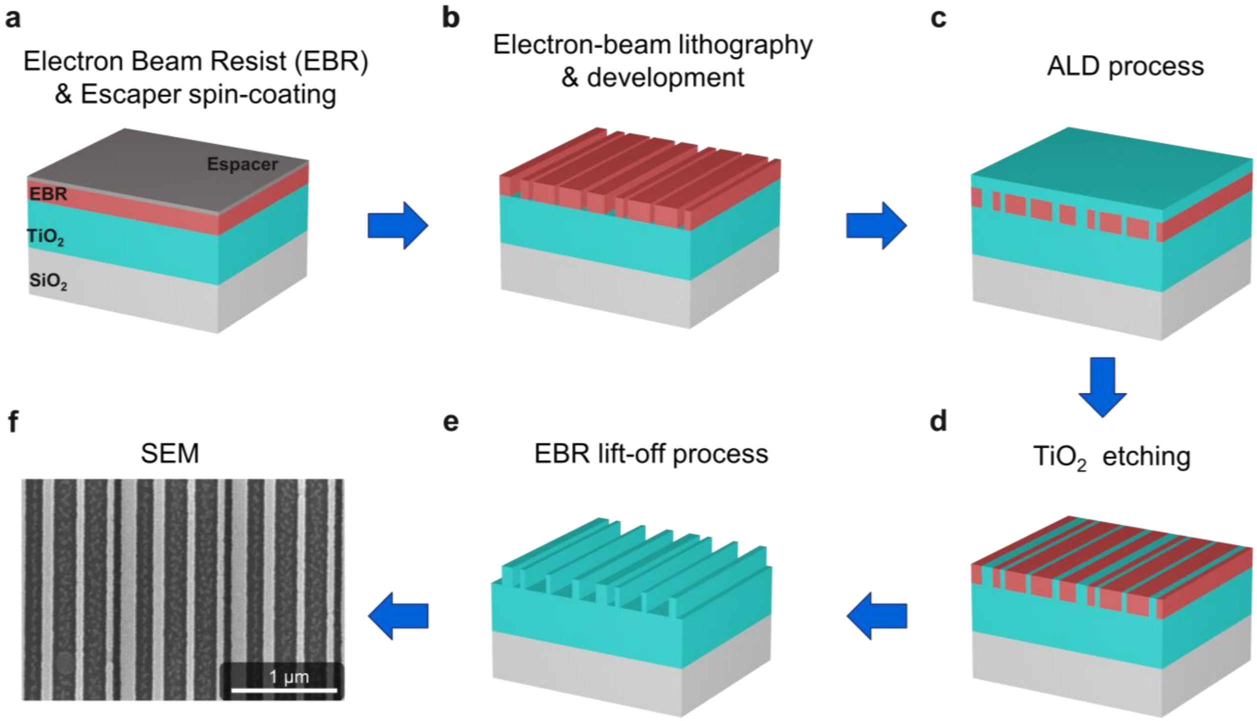

Figure S9. Flowchart of the fabrication process.

S8. Optical setup and experimental results

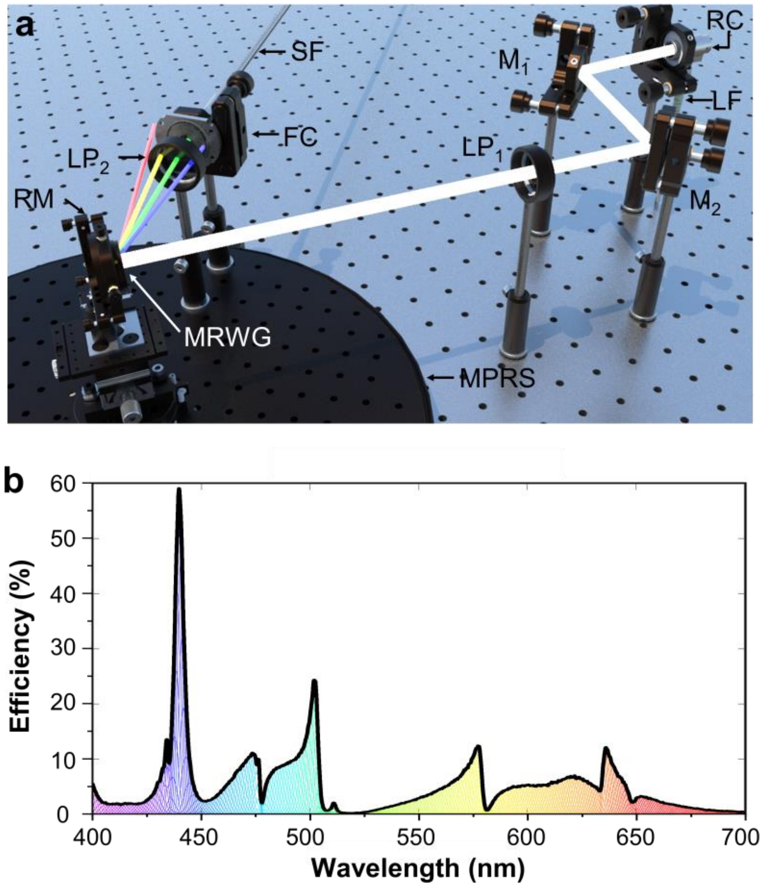

Figure S10. (a) Schematic of the optical setup for angle-dependent diffraction spectrum measurement. LF: laser fiber; RC: reflective collimators; M_1 and M_2: mirror 1 and 2; LP1 and LP2: line polarizer 1 and 2; MPRS: motorized precision rotation stage; MRWG: metasurface resonant waveguide grating sample; RM: rotation mount; FC: fiberport collimators; SF: spectrometer fiber. (b) We conducted measurements using the MPRS with a measurement scale of 0.1 degrees to obtain angle-dependent spectra (color lines). We then extracted the envelope (black line) of the obtained overall first-order reflective diffraction efficiency spectrum. The reference power spectrum used for the efficiency calculation is obtained by measuring the zero-order transmission power through a chromium aperture with the same diameter as the sample. The transmittance of fused silica is also taken into account during the efficiency calculation.

The experimental spectra in Figure S10b show a deviation from the target diffraction wavelengths compared to optimization results (Figure 2b). To identify the fabrication error, we

adjusted some parameters in simulation and compared it with the experimental spectrum shown in Figure 4b. The simulated spectra were calculated using RCWA, considering a lower refractive index for TiO_2 and over-etched waveguides.

Figure S11 shows the comparison of refractive index in design and experiment. The decrease in refractive index is attributed to changes in the ALD conditions after a 2-year process. Additionally, we found that the top grating was under-etched following EBR removal. Consequently, additional etching was performed on the grating and waveguide, resulting in a thinner waveguide in the experiment. We simulated with different waveguide thicknesses to match the spectral peak to our experimental data, thereby determining the actual waveguide thickness. The simulation result shown in Figure 4b is in good agreement with our experimental result, indicating that the waveguide thickness is 344 nm, which corresponds to an over-etching of 60 nm.

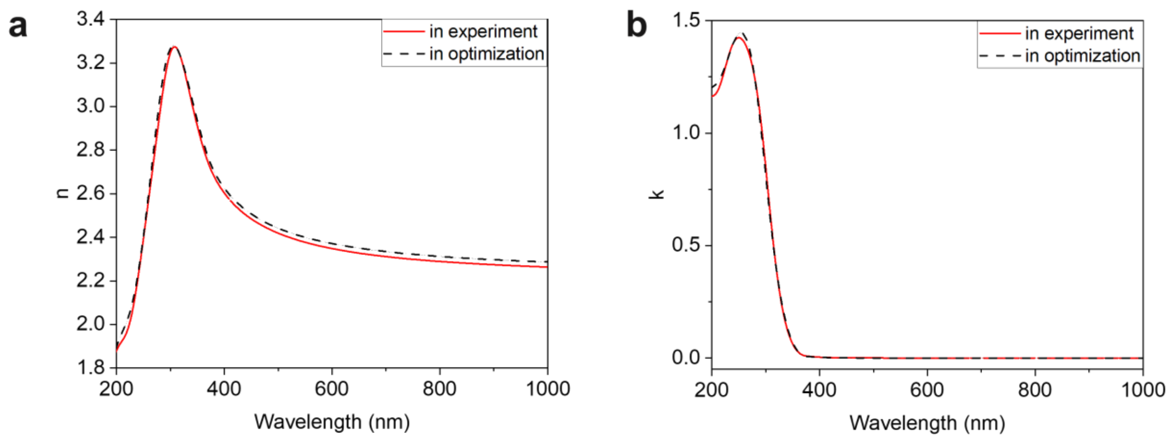

Figure S11. Real part (a) and imaginary part (b) of refractive indexes of TiO_2 deposited by ALD. The optimization one was measured in 2022, which was used in the optimization and design. The experimental one was measured in 2024, which is used to investigate structural and refractive index inaccuracies.

Table S1. Experimental statistics of MRWG.

	Wavelength (nm)	Efficiency (%)	FWHM (nm)	*Q*-factor	Diffraction angle (degree)
λ_1	440	59.1	4.72	93.3	30.36
λ_2	502	24.3	6.63	75.3	35.23
λ_3	577	12.4	12.5	45.6	41.55
λ_4	636	12.1	9.30	69.0	46.96

The Q-factor is defined as $f/\Delta f$, where f is the frequency of the resonance of interest and Δf is the frequency width of the resonance. To obtain the measured Q-factor in Table S1, the frequency width of the resonance Δf is determined by the full width at half maximum of the peak efficiency spectrum (represented by the black envelope line in Figure S10b) in the frequency domain.
